# Spousal concordance of ideal cardiovascular health metrics: findings from the 2014–2019 Korea National Health and Nutrition Examination Survey

**DOI:** 10.1186/s40885-022-00224-3

**Published:** 2022-12-15

**Authors:** Manh Thang Hoang, Hokyou Lee, Hyeon Chang Kim

**Affiliations:** 1 Department of Public Health, Graduate School, Yonsei University, Seoul, Republic of Korea; 2 Department of Preventive Medicine, Yonsei University College of Medicine, Seoul, Republic of Korea; 3 Institute of Health Services Research, Yonsei University College of Medicine, Seoul, Republic of Korea

**Keywords:** Cardiovascular diseases, Spouses, Korea, Epidemiology

## Abstract

**Backgrounds:**

We aimed to investigate whether a spouse’s cardiovascular health (CVH) metrics status affects the other spouse’s ideal CVH using a Korea nationwide representative survey.

**Methods:**

We used the health data of 6,030 married couples who participated in the Korea National Health and Nutrition Examination Survey from 2014 to 2019. The CVH was defined using seven metrics: smoking status, blood pressure, body mass index, total cholesterol, fasting blood glucose, physical activity, and diet, following the American Heart Association guidelines and modifications for body mass index cutoffs and diet quality. The CVH score was calculated on a scale ranging from 0 to 7, with the ideal CVH defined as attaining ideal scores in at least five CVH metrics. Multiple logistic regression analyses were used to assess whether husband’s ideal CVH was associated with his wife’s odds for having ideal CVH, and vice versa.

**Results:**

The mean CVH scores were 3.2 and 4.0 for husband and wife, respectively. After fully adjusting for age and education of both partners and household income, husbands had 1.49 times (95% confidence interval [CI], 1.27–1.69) higher odds of achieving ideal CVH if their wives had also achieved ideal CVH. Meanwhile, wives whose husbands achieved ideal CVH also had 1.46 times (95% CI, 1.27–1.69) higher odds of achieving ideal CVH. Nonsmoking (57.17%), ideal fasting blood glucose level (34.93%), and ideal diet intake (24.18%) were the most concordant CVH metrics among spouses.

**Conclusions:**

Our study found a significant spousal concordance of ideal CVH in Korean married couples. This finding supports the use of a couple-based interventional strategy targeted to promote CVH.

**Supplementary Information:**

The online version contains supplementary material available at 10.1186/s40885-022-00224-3.

## Background

Cardiovascular disease (CVD) is a major contributor to the world’s disease burden and remains the leading global cause of death (18.6 million in 2019) [1, 2], with CVD-associated mortality projected to only increase in the future (> 23.6 million by 2030) [3]. In Korea, the absolute number of deaths engendered by all diseases of the circulatory system has increased from 2009 to 2018 (62,947 deaths in 2018), mostly due to increase in incidence of ischemic heart disease and heart failure [4]. Notably, one in every 40 adults is a stroke patient, and 232 people among 100,000 experience a stroke event every year; among them, 30 individuals die of stroke-related causes [5]. In 2010, the American Heart Association (AHA) defined and emphasized a new concept, ideal cardiovascular health (CVH), which comprises both ideal health behaviors (nonsmoking, body mass index [BMI] < 25 kg/m^2^, physical activity at optimum levels, and pursuit of a diet consistent with current guideline recommendations) and ideal health factors (untreated total cholesterol < 200 mg/dL, untreated blood pressure < 120/80 mmHg, and fasting blood glucose < 100 mg/dL) [6]. This broad, newly introduced concept was adopted to expand the scope of CVD prevention programs and allow interventions to not only focus on reducing CVD incidence but also improve CVH in populations as a whole [6]. Some recent studies show that interaction between spouses positively impacts CVD prevention through behavioral and lifestyle factors [7, 8]. In Korea, previous studies either reported the association of spousal concordance and metabolic syndrome (risks of CVD) [9–11] or presented the trend of CVH metrics from 2000 to 2009 [12]. However, the evidence of the association of spousal concordance regarding ideal CVH in Korea remains limited. In 2016, a systematic review conducted by Younus found only two papers related to CVH in Korea [13]. Moreover, these studies in Korea investigated the epidemiology of metabolic syndrome to identify effective strategies for CVD prevention [10, 11]. Since the diagnostic criteria for metabolic syndrome does not include lifestyle factors, these studies could not explore the concordance of behavioral factors independently or in conjunction with health factors among couples. The present study aims to address this research gap and uses Korean national data to find evidence of the spousal concordance in ideal CVH in the Korean population.

## Methods

### Ethical statements

Korea National Health and Nutrition Examination Survey (KNHANES) is administered by the Korea Centers for Disease Control and Prevention (KCDC) and approved by the Institutional Review Board of KCDC. Every participant gave their informed consent in writing before participating in the study.

### Study population

KNHANES is a large household survey conducted on a national scale to examine the general health and nutritional status of Korean people. It consists of basic household interviews, health interviews, physical examinations, and nutrition-related surveys. Participants are randomly selected from the sampled household unit using a stratified multistage probability sampling design based on geographical area, sex, and age group using a household census from the National Census Registry in Korea. Details of the survey method are reported elsewhere [14, 15]. From 2014 to 2019, 74,662 individuals aged at least 20 years participated in the survey and completed the health examinations. Among them, 11,081 married couples finished the examination and were included in the analyses. We further excluded 1,006 couples with at least one partner diagnosed with CVD, 1,544 couples with one partner who had not fasted for ≥ 8 h before blood sample collection, and 107 couples with pregnant wives. Additionally, one couples were excluded because one person had extreme energy intake and 2,393 couples were removed because their data for at least one CVH metric was missing. Finally, 6,030 couples were included in the analyses.

### Definition of cardiovascular health

The following seven components of CVH were evaluated: smoking status, blood pressure, BMI, total cholesterol, fasting blood glucose, physical activity, and diet [6, 8]. For blood pressure measurements, participants underwent a standard exam using a mercury sphygmomanometer and an appropriate-sized cuff to measure blood pressure on the right arm after 5 min of rest. Three measurements were taken and the average of all three readings was used in the analysis. Each CVH metric was classified as ideal, intermediate, or poor according to the AHA guidelines [6], except for BMI and healthy diet score. For BMI, to appropriately account for Asian populations and the World Health Organization recommendations, we coded BMI as an ideal group (if BMI < 23 kg/m^2^), intermediate group (23 kg/m^2^ ≤ BMI < 25 kg/m^2^) and poor group (BMI ≥ 25 kg/m^2^) [16]. For healthy diet score, because of the difference in diet measurement tool in AHA guideline those in the food frequency questionnaire of KNHANES, we used a healthy diet score that was developed by another previous study and based on the guideline for the Dietary Approaches to Stop Hypertension and modified by the Korean diet guideline for dyslipidemia [12, 17]. A healthy diet met the following five criteria: it ensured a total daily fat intake < 35% of total calories, total daily protein intake > 15% of total calories, total daily carbohydrate intake < 55% of total calories, total daily sodium intake < 2,300 mg, and total daily fiber intake > 20 g. We dichotomized the healthy diet score variable and classified each score as either ideal (met at least three criteria) or poor (met fewer than three criteria). The details are available in Table S1.

We recoded the seven metrics as dichotomous variables, granting 1 point for the ideal category versus 0 point for the other categories, and then calculated the CVH score as the sum of all seven metrics; the CVH score ranged from 0 to 7. In our study, ideal CVH was attained when the CVH score was ≥ 5, indicating that the participant achieved ideal scores in at least five individual CVH metrics.

### Statistical analyses

#### Primary analysis

The characteristics of the study population were obtained from descriptive statistics and demonstrated using dyads. Continuous variables are presented as means with standard deviations. Spearman correlation and correlation adjusted by age of both husband and wife were calculated and presented by dyads. Prevalence for husband and wife CVH score or category by his or her partner CVH score category was calculated. We also tabulated the number and percentage of concordant and discordant dyads.

We examined the association of each participant’s CVH with those of their spouses using logistic regression to estimate the odds ratio for the binary outcomes (ideal CVH vs. nonideal CVH) and two types of exposures (category CVH score and continuous CVH score). The association was adjusted for the ages, education level of both partners, and household income.

#### Secondary analysis

First, to further determine whether significant associations of the CVH of couples were driven by any single CVH metric, we repeated the analyses using individual CVH metrics of each partner (husband/wife) as an outcome (ideal status vs. nonideal status) and the individual CVH metrics of their partner as an exposure (ideal status vs. nonideal status). Second, to distinguish the contribution of health factors and behavioral factors to significant spousal concordance and CVH association, we repeated logistic regression models for behavioral outcomes (including physical activity, diet, BMI, and smoking) and health outcomes (blood pressure level, total cholesterol level, and fasting blood glucose level) (Table S2). Third, to examine the influence of age on the relationship between the CVH of partners in a couple, we stratified the primary analysis by the tertile of the average age between spouses (Fig. 3).

#### Sensitivity analysis

Considering the selection bias when applying the exclusion criteria and the impact of missing data, we calculated the descriptive statistics of the original couple population (Table S3). In addition, we conducted a sensitivity analysis for the primary spousal concordance and CVH association, not excluding participants with missing values for any CVH metric. The missing value among the seven metrics of CVH was coded as 0 when calculating the CVH score (Table S4). All analyses were performed using SAS ver. 9.4 (SAS Institute Inc., Cary, NC, USA).

## Results

### Study participants

**Fig. 1:**
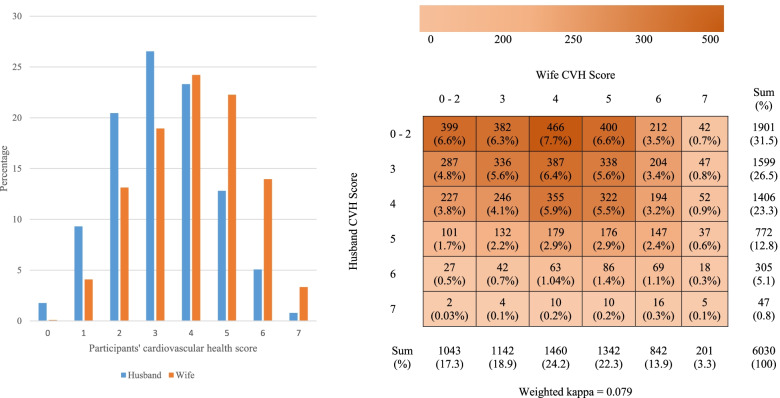
Frequencies and percentages of husband’s cardiovascular health score and wife’s cardiovascular health score

Table 1 presents participants’ characteristics as per sex and spousal correlations in 6,030 married Korean couples. All characteristics were highly correlated between the spouses, even after adjusting for the age of both spouse. Husbands were approximately 3 years older (54.3 ± 13.4 years) than their wives (51.2 ± 12.9 years) on average. Wives tended to have lower BMI, fasting blood glucose levels, triglyceride levels, and blood pressure, as well as higher high-density lipoprotein cholesterol levels. The mean ± standard deviation of CVH score was 3.2 ± 1.4 out of 7 among husbands and 18.7% of husbands achieved ideal CVH. The mean ± standard deviation of CVH score was 4.0 ± 1.5 out of 7 among wives, which is higher than the mean CVH score among husbands, and 39.5% of wives achieved ideal CVH. Most of the husbands (26.5%) attained three ideal CVH metrics, whereas most wives (24.2%) attained four ideal CVH metrics (Table 1, Fig. 1).

**Table 1:** Characteristic of study participants

Characteristic	Husband (*n* = 6,030)	Wife (*n* = 6,030)	Correlation		Age-adjusted correlation	
			Coefficient	*P*-value	Coefficient	*P*-value
Age (yr)	54.3 ± 13.4	51.2 ± 12.9	0.971	< 0.001	-	-
Weight (kg)	71.3 ± 10.9	58.4 ± 8.9	0.067	< 0.001	0.071	< 0.001
Waist circumference (cm)	87.2 ± 8.3	79.3 ± 9.1	0.105	< 0.001	0.107	< 0.001
BMI (kg/m^2^)	24.6 ± 3.1	23.5 ± 3.4	0.041	0.001	0.082	< 0.001
Fasting blood glucose (mg/dL)	104.8 ± 24.2	98.1 ± 21.1	0.149	< 0.001	0.092	< 0.001
HbA1c (%)	5.82 ± 0.85	5.68 ± 0.72	0.209	< 0.001	0.078	< 0.001
Total cholesterol (mg/dL)	192.7 ± 36.3	195.3 ± 36.2	0.053	< 0.001	0.081	< 0.001
HDL-cholesterol (mg/dL)	46.9 ± 11.1	54.5 ± 12.3	0.082	< 0.001	0.077	< 0.001
LDL-cholesterol (mg/dL)	116.6 ± 32.2	118.5 ± 32.1	0.056	< 0.001	0.076	< 0.001
Triglyceride (mg/dL)	163.3 ± 131.1	114.7 ± 75.4	0.063	< 0.001	0.099	< 0.001
Systolic blood pressure (mmHg)	121.6 ± 14.8	116.9 ± 17.2	0.205	< 0.001	0.086	< 0.001
Diastolic blood pressure (mmHg)	78.3 ± 9.9	74.3 ± 9.4	0.075	< 0.001	0.112	< 0.001
Total energy intake (kcal)	2,364.7 ± 931.8	1,713.5 ± 677.6	0.245	< 0.001	0.221	< 0.001
Fat intake (g)	50.3 ± 36.8	37.5 ± 26.4	0.423	< 0.001	0.335	< 0.001
Protein intake (g)	84.3 ± 42.9	61.2 ± 30.4	0.315	< 0.001	0.279	< 0.001
Carbohydrate intake (g)	346.4 ± 128.0	274.3 ± 113.2	0.241	< 0.001	0.246	< 0.001
Sodium intake (mg)	4,322.7 ± 2,504.9	3,073.5 ± 1,919.5	0.299	< 0.001	0.276	< 0.001
Fiber intake (g)	29.0 ± 14.4	24.9 ± 14.3	0.356	< 0.001	0.346	< 0.001
Total CVH score	3.2 ± 1.4	4.0 ± 1.5	0.138	< 0.001	0.122	< 0.001

Data are presented as mean ± standard deviation

*BMI* Body mass index, *HbA1C* Hemoglobin A1C, *HDL* High-density lipoprotein, *LDL* Low-density lipoprotein

**Fig. 2:**
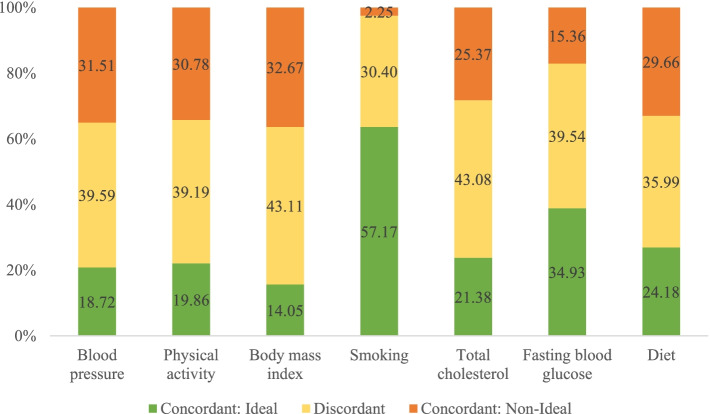
Percentage of husband/wife concordance or discordance by the ideal cardiovascular health metrics

Figure 2 shows the proportion of couples whose findings were concordant for each ideal CVH variable. Concordance in CVH metrics was categorized as follows: both partners received an ideal status, both partners received a nonideal status, or the status was discordant for the respective variable. Most of the spouses’ metric were concordant for individual CVH variables, with total concordance rates greater than 50%. The highest concordance in achieving individual ideal CVH metric were observed for nonsmoking (57.2%), followed by ideal fasting blood glucose level (34.9%) and ideal diet intake (24.2%).

### Primary analysis

Husbands had 1.49 times higher adjusted odds of achieving ideal CVH if their partner also achieved ideal CVH (95% confidence interval [CI], 1.27–1.69). Wives also had 1.46 times higher adjusted odds of having ideal CVH if their husbands also did (95% CI, 1.27–1.69) (Table 2). This indicated that participants whose spouses had a higher CVH score had a higher chance of attaining an ideal CVH than those whose partners had a lower CVH score. The adjusted odds ratios (ORs) ranged from 1.08 to 2.46 when their spousal CVH score increased and were mostly significant. Significantly adjusted OR per 1 CVH score of 1.18 and 1.14 were found respectively for husbands and wives as outcome (Table 2).

**Table 2:** Odds ratio of a participant having ideal CVH according to their spousal CVH

CVH metric	No. of wives	Husbands with ideal CVH (score ≥ 5)		No. of husbands	Wives with ideal CVH (score ≥ 5)	
		No. (%)	Odds ratio (95% CI)^a)^		No. (%)	Odds ratio (95% CI)^a)^
Spousal CVH metrics score						
0–2	1,043	130 (12.5)	1.00	1,901	654 (34.4)	1.00
3	1,142	178 (15.6)	1.25 (0.97–1.59)	1,599	589 (36.8)	1.08 (0.93–1.26)
4	1,460	252 (17.3)	1.33 (1.05–1.68)	1,406	578 (41.1)	1.28 (1.09–1.49)
5	1,342	272 (20.3)	1.53 (1.19–1.95)	772	360 (46.6)	1.44 (1.19–1.74)
6	842	232 (27.6)	2.20 (1.70–2.86)	305	173 (56.7)	2.03 (1.54–2.66)
7	201	60 (29.9)	2.36 (1.63–3.43)	47	31 (65.9)	2.46 (1.28–4.73)
Per 1 higher (continuous)	-	-	1.18 (1.13–1.25)	-	-	1.14 (1.09–1.18)
Spousal CVH metrics status						
Nonideal	3,645	560 (15.4)	1.00	4,906	1,821 (37.1)	1.00
Ideal	2,385	564 (23.7)	1.49 (1.27–1.69)	1,124	564 (50.2)	1.46 (1.27–1.69)

CVH, cardiovascular health; CI, confidence interval

^a ^Adjusted for age and education of both partners and household income

### Secondary analysis

Although spousal concordance was observed in all seven metrics of the CVH, there were differences in the concordance’s strength among metrics. The ideal smoking status showed the highest spousal concordance (adjusted ORs [95% CI] were 3.77 [2.71–5.26] and 3.73 [2.67–5.21] in husbands and wives as outcome, respectively), followed by the ideal diet status (adjusted ORs [95% CI] were 2.18 [1.97–2.43] in both husbands and wives as outcome). Furthermore, the odds of concordances in the remaining five metrics ranged from 1.21 to 1.55 (Table 3). After dichotomizing CVH measurements into behavioral factors and health factors, we found positively significant association between husbands’ CVH status and wives’ CVH status. The strength of the concordance contributed by behavioral factors was minimally greater than that contributed by health factors (Table S2).

**Table 3:** Spousal concordance of each CVH metric in 6,030 married couples

CVH metric	Wife’s condition	No. of wives (column %)	Husbands with ideal condition		Husband’s condition	No. of husbands (column %)	Wives with ideal condition	
			No. (%)	Odds ratio (95% CI)^a)^			No. (%)	Odds ratio (95% CI)^a)^
Blood pressure	Nonideal	2,855 (47.3)	740 (25.9)	1.00	Nonideal	4,034 (66.9)	1,919 (47.6)	1.00
	Ideal	3,176 (52.7)	1,257 (39.6)	1.21 (1.07–1.38)	Ideal	1,997 (33.1)	1,257 (62.9)	1.23 (1.08–1.39)
Physical activities	Nonideal	3,536 (58.6)	1,470 (41.6)	1.00	Nonideal	3,228 (53.5)	1,162 (36.0)	1.00
	Ideal	2,495 (41.4)	1,333 (53.4)	1.54 (1.39–1.72)	Ideal	2,803 (46.5)	1,333 (47.6)	1.55 (1.39–1.72)
Body mass index	Nonideal	3,065 (50.8)	871 (28.4)	1.00	Nonideal	4,217 (69.9)	2,023 (47.9)	1.00
	Ideal	2,966 (49.2)	943 (31.8)	1.35 (1.20–1.52)	Ideal	1,814 (30.1)	943 (51.9)	1.35 (1.21–1.52)
Smoking	Nonideal	203 (3.4)	52 (25.6)	1.00	Nonideal	2,140 (35.5)	1,989 (92.9)	1.00
	Ideal	5,828 (96.6)	3,839 (65.9)	3.77 (2.71–5.26)	Ideal	3,891 (64.5)	3,839 (98.7)	3.73 (2.67–5.21)
Total cholesterol	Nonideal	3,217 (53.3)	1,514 (47.1)	1.00	Nonideal	3,082 (51.1)	1,379 (44.7)	1.00
	Ideal	2,814 (46.7)	1,435 (51.0)	1.25 (1.12–1.39)	Ideal	2,949 (48.9)	1,435 (48.7)	1.25 (1.12–1.39)
Fasting blood glucose	Nonideal	1,817 (30.1)	786 (43.3)	1.00	Nonideal	2,900 (48.1)	1,869 (64.5)	1.00
	Ideal	4,214 (69.9)	2,345 (55.6)	1.30 (1.16–1.47)	Ideal	3,131 (51.9)	2,345 (74.9)	1.32 (1.17–1.48)
Diet	Nonideal	3,254 (53.9)	1,263 (38.8)	1.00	Nonideal	3,144 (52.1)	1,153 (36.7)	1.00
	Ideal	2,777 (46.1)	1,624 (58.5)	2.18 (1.97–2.43)	Ideal	2,887 (47.9)	1,624 (56.3)	2.18 (1.97–2.43)

*CVH* Cardiovascular health, *CI* Confidence interval

^a ^Adjusted for age and education of both partners and household income

**Fig. 3:**
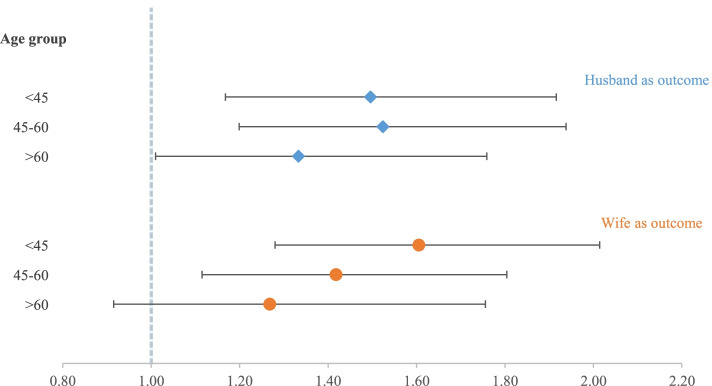
Odds ratio of a participant achieving ideal cardiovascular health if whose partner does, by age. Odds ratios were adjusted for age and education of both partners, household income

On examining the spousal concordance of attaining ideal CVH in different age groups, we observed a tendency for spousal concordance ORs decreased in wives when ages increased (Fig. 3). However, among husbands no such tendency was observed in the ORs, but the spousal correlations were significant in all three groups when investigating the effect of the wife’s CVH status on the husband’s CVH status. In addition, we found a strong overlap of 95% CIs of the corresponding ORs in husbands, which might claim a persistent influence of the wife’s CVH on the husband’s CVH when they get older.

### Sensitivity analysis

Among the original couples that we found from our data (11,082 married couples), the characteristics of the husbands and wives in Table S3 did not significantly differ from those of the study participants in Table 1. Besides, after including participants with missing value for any CVH metric and recoding those missing value as 0, the associations between spousal concordance and ideal CVH in the sensitivity analysis were robust compared to those in the primary analysis, with some strengthening of point estimates but overlapping confidence intervals (Table S4).

## Discussion

The primary finding of this study was that CVH was significantly concordant among spouses in 6,030 married couples from Korea. The nonsmoking status showed the highest spousal concordance, following by the ideal diet status. The results were generally consistent across primary, secondary and sensitivity analysis. In the age-stratified analysis, a tendency for spousal concordance ORs decreased when ages increased was found when investigating the effect of the husbands’ CVH status on their wives’ CVH status.

Previously, several studies used various threshold levels to classify the ideal CVH. Folsom et al. [18] defined a score of 10 to 14 as optimal, 5 to 9 as average, and 0 to 4 as inadequate for CVH. Desai et al. [19] grouped participants with point scores ≤ 8 as having poor CVH, those with scores of 9 to 11 as having intermediate CVH, and those with scores ≥ 12 as having ideal CVH. The common factor in these studies is that each individual metric was set as a score of 0 (poor), 1 (intermediate), or 2 (ideal); then, the ideal CVH was classified on the basis of the total score. However, the ideal CVH derived from the total CVH scores, which were calculated through these methods, can be biased from the contribution of intermediate metrics. To account for that bias, in our study, achieving the ideal CVH required achieving ideal scores in at least five individual CVH metrics. We also found some other articles with approaches similar to ours [20, 21].

Consistent with other studies in Korea, our study demonstrated a low prevalence of ideal CVH in the community [12, 22]. Similar to previous studies, our findings showed strong spousal concordance for CVH and its metrics [7, 8]. Among the seven metrics evaluated in our study, factors that were most likely to be concordant among couples were nonsmoking and ideal diet. Similar results have been reported in prior studies [7, 8]. When compared with previous articles in Korea, we also observed significant spousal concordance for metabolic syndrome and its components [10, 11]. Even though the parameters for measuring metabolic syndrome and CVH differ, our results showed a great mutual influence within a couple, which may affect CVD prevention. However, metabolic syndrome mainly focuses on clinical indicators (or health factors in CVH). Contrastingly, CVH includes the impact of behavioral factors, and according to our outcomes, the spouses’ behavioral factors outweighed health factors in contributing to achieving ideal CVH. The high concordance of nonsmoking and ideal diet among couples can partly explain this finding. Scientists have found that smoking has been linked to other clinical indicators in CVH metrics, such as lowering high-density lipoprotein cholesterol, causing thickening and narrowing of blood vessels [23]. Meanwhile, the diet has contributed to BMI, blood glucose level, and total cholesterol level. Our observations suggest that public health intervention programs should increasingly focus on lifestyle modification.

Our findings are particularly important because ideal CVH are shown to predict not only the future risk of CVD incidence but also all-cause mortality [13, 24–27]. According to a systematic review, numerous prior studies have reported spousal concordance for several cardiovascular risk factors [7, 9–11]. Cohabitation factors are believed to play a role in the observed spousal correlations. Our study, as well as various other studies, have demonstrated the association between people’s health and their partner’s health behavior [7–9]. In general, spouses share common environmental factors, which implies the sharing of many aspects of lifestyle.

Our results suggest that in the future, couple-based interventions that target healthy behaviors can be effective methods for preventing CVD events. Even in individual-based interventions, nontargeted members of a couple may be benefited because of the strong couple concordance in behavior factors. For instance, enhancing dyadic efficacy for smoking cessation may be a promising approach in couple-based prevention interventions [28, 29].

It is necessary to further analyze the significant association between spousal concordance and CVH when stratifying by age group. In the same age strata, the odd of one partner having an ideal CVH when the other partner also had an ideal CVH was similar when considering either the husband’s CVH or the wife’s CVH as the outcome. The same result was observed in the analysis without age stratification (Table 2). However, an interesting pattern of reduction was seen in the ORs when investigating the effect of the husbands’ CVH status on the wives’ CVH status with increasing age. It is well known that higher age is probably associated with longer marriage duration, which motivated us to check whether CVH score concordance between couples was influenced by the duration of marriage. Fig. S1 shows that the length of marriage similarly affected the husband and wife, with the same pattern and overlapping 95% CI. We also tested the interaction of partner CVH and marriage duration in the covariate-adjusted model; however, we did not find a significant interaction (Fig. S1). Combining all the above findings, we can hypothesize that apart from cohabitant factors, there are other factors that only or differently affect women. One explanation might be the presence of menstruation and menopause, which women normally experience during middle age. The AHA highlighted the complexity of menopause transition and its relevance to critically adverse cardio-metabolic health changes that are independent of chronological aging [30]. Another explanation could be the intriguing relationship between mental health disorders and CVD. The potential mechanism of this phenomenon is that certain mental health disorders may trigger physical changes (less likely to get regular exercise or more likely to drink too much alcohol) that can elevate heart risk in several ways [31–33]. We did not measure the prevalence of depression or mental disorder in our dataset, but major depressive disorder is more prevalent among Korean women than Korean men [34].

Our study has several limitations. First, the study design was cross-sectional, which limited our ability to interpret causal relations in our findings. Second, removing any participant with missing value for any CVH metric may have led to selection bias. However, we tried to account for this bias by comparing our study sample’s descriptive statistics with those of the total couple population (11,081 dyads) and performing a sensitivity analysis. Third, we could not measure marital satisfaction, which is suggested to be associated with health outcomes at a later date [35]. Diet and exercise data were drawn from questionnaires, which could overestimate ideal diet and exercise patterns because individuals are known to be overoptimistic in self-reporting such traits. In addition, as mentioned previously, the criteria for a healthy diet are arbitrary and do not completely correspond with the AHA’s definition of a healthy diet. However, to the best of our knowledge, we adapted other available guidelines to the existing KNHANES data and fit them with CVH metrics. Finally, although we carefully controlled for major known confounders, the findings may be partly explained by differences in unknown confounders. In addition, socioeconomic status (represented by education and household income in our study) may be a significant mediator of the study finding because family members usually belong to the same socioeconomic class, which is a powerful predictor of health behavior. Further investigation is needed in future.

Despite the limitations of this study, there are strengths that merit consideration when interpreting the results. First, we explored the association of ideal CVH as a construct among couples in Korea. The findings corroborate those of other studies and can help identify approaches to preventative interventions that can help achieve the goal of the National Health Plan for Cardiovascular Disease in Korea. Second, the study sample was large (6,030 couples) and nationally representative. Therefore, this study had sufficient power to explore the prevalence and strength of spousal concordance in ideal CVH and to extrapolate the findings to the general Korean population.

## Conclusions

In this study of Korean married couples, we observed significant interspousal concordance in CVH. The development and testing of dyadic interventions promoting ideal CVH are highly recommended. Interventional programs targeted toward improving lifestyle behaviors among married couples would likely benefit both partners.

## Supplementary Information

**Additional file 1: Table S1.** Cardiovascular health metrics using AHA criteria for adults aged ≥20 years. **Table S2.** Odds ratio of a participant having ideal cardiovascular health according to the corresponding cardiovascular health of their spouse, among 6030 married couples. **Table S3.** Characteristic of 11,082 married couples. **Table S4.** Odds ratio of a participant having ideal cardiovascular health according to the cardiovascular health score/status of their spouse, among 8,423 married couples. **Fig. S1.** Odds ratio of a participant achieving ideal cardiovascular health if whose partner also does, by marriage duration.

## Data Availability

The datasets generated and/or analyzed during the current study are available in the Korea National Health and Nutrition Examination Survey repository (https://knhanes.kdca.go.kr/knhanes/sub03/sub03_02_05.do).

## Abbreviations

AHA: American Heart Association
BMI: Body mass index
CI: Confidence interval
CVD: Cardiovascular disease
CVH: Cardiovascular health
HbA1C: Hemoglobin A1C
HDL: High-density lipoprotein
KCDC: Korea Centers for Disease Control and Prevention
KNHANES: Korea National Health and Nutrition Examination Survey
LDL: Low-density lipoprotein
OR: Odds ratio
