# Supplementary material for: Spousal concordance of ideal cardiovascular health metrics: findings from the 2014–2019 Korea National Health and Nutrition Examination Survey
Source: Clin Hypertens. 2022 Dec 15;28:41. doi: 10.1186/s40885-022-00224-3 (PMC9753352; doi:10.1186/s40885-022-00224-3)
Supplement: Supplementary file 1 — Additional file 1: Table S1. Cardiovascular health metrics using AHA criteria for adults aged ≥20 years. Table S2. Odds ratio of a participant having ideal cardiovascular health according to the corresponding cardiovascular health of their spouse, among 6030 married couples. Table S3. Characteristic of 11,082 married couples. Table S4. Odds ratio of a participant having ideal cardiovascular health according to the cardiovascular health score/status of their spouse, among 8,423 married couples. Fig. S1. Odds ratio of a participant achieving ideal cardiovascular health if whose partner also does, by marriage duration. [file 40885_2022_224_MOESM1_ESM.docx]

**Table S1.** Cardiovascular health metrics using AHA criteria for adults aged ≥20 years

| Goal/metric | Source | Poor health  (0 point) | Intermediate health  (0 point) | Ideal health  (1 point) |
| --- | --- | --- | --- | --- |
| Blood pressure | AHA | SBP ≥140 mmHg or DBP ≥90 mmHg | SBP 120–139 mmHg or DBP 80–89 mmHg or treated to goal | <120/<80 mmHg |
| Physical activity | AHA | None | 1–149 min/wk moderate intensity or 1–74 min/wk vigorous intensity or 1–149 min/wk moderate + vigorous | ≥150 min/wk moderate intensity or ≥75 min/wk vigorous intensity or ≥150 min/wk moderate + vigorous |
| Body mass index | WHO recommendation for Asia population | ≥25 kg/m^2^ | 23–24.9 kg/m^2^ | <23 kg/m^2^ |
| Current smoking | AHA | Yes | Former ≤12 mo | Never or quit >12 mo |
| Total cholesterol | AHA | ≥240 mg/dL | 200–239 mg/dL or treated to goal | <200 mg/dL |
| Fasting blood glucose | AHA | ≥ 126 mg/dL | 100–125 mg/dL or treated to goal | <100 mg/dL |
| Healthy diet | Modified following the Dietary Approaches to Stop Hypertension and the Korean diet guideline for dyslipidemia | 0–2 components |  | 3–5 Components  Fat intake <35% total energy intake  Protein >15% total energy intake  Carbohydrate <55% total energy intake  Sodium <2,300 g  Fiber >20 g |

AHA, American Heart Association; SBP, Systolic blood pressure; DBP, diastolic blood pressure; WHO, World Health Organization.

**Table S2.** Odds ratio of a participant having ideal cardiovascular health according to the corresponding cardiovascular health of their spouse, among 6,030 married couples

| Spousal cardiovascular health metric | No. of  wives | Husbands with ideal condition^a)^ | | No. of  husbands | Wives with ideal condition^a)^ | |
| --- | --- | --- | --- | --- | --- | --- |
|  |  | No. (%) | Odds ratio (95% CI) |  | No. (%) | Odds ratio (95% CI) |
| Behavioral factors^b)^ |  |  |  |  |  |  |
| Nonideal | 3,466 | 796 (22.9) | 1.0 | 4,397 | 1,727 (39.3) | 1.0 |
| Ideal | 2,564 | 837 (32.6) | 1.57 (1.39–1.77) | 1,633 | 837 (51.3) | 1.57 (1.39–1.76) |
| Health factors^c)^ |  |  |  |  |  |  |
| Nonideal | 2,536 | 896 (35.3) | 1.0 | 3,476 | 1,836 (52.8) | 1.0 |
| Ideal | 3,494 | 1,658 (47.5) | 1.27 (1.12–1.43) | 2,554 | 1,658 (64.9) | 1.28 (1.14–1.45) |

Odds ratios were adjusted for age and education of both partners and household income.

CI, confidence interval.

^a)^Ideal condition means participants either having ideal behavioral factors status or ideal health factors status; ^b)^Behavioral factors include physical activity, diet, smoking and body mass index. Nonideal status means total score equals 2 or less. Ideal status means total score equal 3 or 4; ^c)^Health factors include blood pressure status, fasting blood glucose status and cholesterol level. Nonideal status means total score equals 1 or less. Ideal status means total score equal 2 or 3.

**Table S3.** Characteristic of 11,082 married couples

| Characteristic | Husband | |  | Wife | |
| --- | --- | --- | --- | --- | --- |
|  | No. | Mean ± SD |  | No. | Mean ± SD |
| Age (yr) | 11,082 | 55.3 ± 14 |  | 11,082 | 52.2 ± 13.5 |
| Weight (kg) | 10,450 | 70.8 ± 11.1 |  | 10,612 | 58.6 ± 9.2 |
| Waist circumference (cm) | 10,430 | 87.1 ± 8.6 |  | 10,582 | 79.9 ± 9.4 |
| Body mass index (kg/m^2^) | 10,444 | 24.5 ± 3.1 |  | 10,601 | 23.6 ± 3.5 |
| Fasting blood glucose (mg/dL) | 10,047 | 106.1 ± 26.2 |  | 10,159 | 98.9 ± 22.4 |
| Hemoglobin A1c (%) | 10,032 | 5.9 ± 0.9 |  | 10,123 | 5.7 ± 0.7 |
| Total cholesterol (mg/dL) | 10,047 | 190.3 ± 37.3 |  | 10,159 | 194.8 ± 37 |
| HDL-cholesterol (mg/dL) | 10,045 | 46.9 ± 11.2 |  | 10,156 | 54.2 ± 12.6 |
| LDL-cholesterol (mg/dL) | 10,046 | 114.3 ± 32.8 |  | 10,156 | 117.7 ± 32.7 |
| Triglyceride (mg/dL) | 10,047 | 163.69 ± 130.93 |  | 10,159 | 117.27 ± 77.63 |
| Systolic blood pressure (mmHg) | 10,413 | 122.2 ± 15.1 |  | 10,594 | 117.5 ± 17.3 |
| Diastolic blood pressure (mmHg) | 10,413 | 77.8 ± 10.2 |  | 10,594 | 74.1 ± 9.5 |
| Total energy intake (kcal) | 9,509 | 2300 ± 946.7 |  | 10,312 | 1,695.7 ± 679.1 |
| Fat intake (g) | 9,509 | 48 ± 37.5 |  | 10,312 | 36.5 ± 26.6 |
| Protein intake (g) | 9,509 | 81.5 ± 47.4 |  | 10,312 | 60.1 ± 30.2 |
| Carbohydrate intake (g) | 9,509 | 340.1 ± 126.4 |  | 10,312 | 273.2 ± 112.9 |
| Sodium intake (mg) | 9,509 | 4235.9 ± 3010.7 |  | 10,312 | 3,066.8 ± 1,952.2 |
| Fiber intake (g) | 9,509 | 28.4 ± 14.6 |  | 10,312 | 24.5 ± 14.1 |

SD, standard deviation; HDL, high-density lipoprotein; LDL, low-density lipoprotein.

**Table S4.** Odds ratio of a participant having ideal CVH according to the CVH score/status of their spouse, among 8,423 married couples

| CVH metric | No. of  wives | Husbands with ideal CVH (score ≥5) | | No. of  husbands | Wives with ideal CVH  (score ≥5) | |
| --- | --- | --- | --- | --- | --- | --- |
|  |  | No. (%) | Odds ratio  (95% CI)^a)^ |  | No. (%) | Odds ratio  (95% CI)^a)^ |
| Spousal CVH score |  |  |  |  |  |  |
| 0­–2 | 1,684 | 172 (10.2) | 1.0 | 3,158 | 1,030 (32.6) | 1.0 |
| 3 | 1,665 | 222 (13.3) | 1.23 (0.97–1.53) | 2,169 | 764 (35.2) | 1.07 (0.93–1.22) |
| 4 | 1,940 | 303 (15.6) | 1.38 (1.11–1.71) | 1,753 | 694 (39.6) | 1.27 (1.11–1.46) |
| 5 | 1,792 | 320 (17.9) | 1.56 (1.25–1.95) | 935 | 420 (44.9) | 1.43 (1.20–1.69) |
| 6 | 1,089 | 259 (23.8) | 2.24 (1.77–2.85) | 359 | 194 (54.0) | 1.98 (1.54–2.55) |
| 7 | 253 | 67 (26.5) | 2.51 (1.77–3.56) | 49 | 32 (65.3) | 2.51 (1.34–4.73) |
| Per 1 higher (continuous) |  |  | 1.19 (1.14–1.25) |  |  | 1.13 (1.09–1.17) |
| Spousal CVH status |  |  |  |  |  |  |
| Nonideal | 5,289 | 697 (13.2) | 1.0 | 7,080 | 2,488 (35.1) | 1.0 |
| Ideal | 3,134 | 646 (20.6) | 1.48 (1.29–1.69) | 1,343 | 646 (48.1) | 1.46 (1.28–1.67) |

CVH, cardiovascular health; CI, confidence interval.

^a)^Adjusted for age and education of both partners and household income.

P for interaction: 0.9998

P for interaction: 0.4129

**Fig. S1.** Odds ratio of a participant achieving ideal cardiovascular health if whose partner also does, by marriage duration. Odds ratio is adjusted for age, education of both partners, and household income. Interaction term was exposure × marriage duration.
